# Cardiovascular Surgery Residency Program: Training Coronary Anastomosis Using the Arroyo Simulator and UNIFESP Models

**DOI:** 10.5935/1678-9741.20150058

**Published:** 2015

**Authors:** Miguel Angel Maluf, Walter José Gomes, Ademir Massarico Bras, Thiago Cavalcante Vila Nova de Araújo, André Lupp Mota, Caio Cesar Cardoso, Rafael Viana dos S. Coutinho

**Affiliations:** 1 Escola Paulista de Medicina da Universidade Federal de São Paulo (EPM-UNIFESP), São Paulo, SP, Brazil.; 2 Universidade Federal da Bahia (UFBA), Bahia, BA, Brazil.

**Keywords:** Anastomosis, Surgical, Intra-Aortic Balloon Pump, Cardiopulmonary Bypass

## Abstract

**OBJECTIVE:**

Engage the UNIFESP Cardiovascular Surgery residents in coronary anastomosis, assess their skills and certify results, using the Arroyo Anastomosis Simulator and UNIFESP surgical models.

**METHODS:**

First to 6^th^ year residents attended a weekly program of technical training in coronary anastomosis, using 4 simulation models: 1. Arroyo simulator; 2. Dummy with a plastic heart; 3. Dummy with a bovine heart; and 4. Dummy with a beating pig heart. The assessment test was comprised of 10 items, using a scale from 1 to 5 points in each of them, creating a global score of 50 points maximum.

**RESULTS:**

The technical performance of the candidate showed improvement in all items, especially manual skill and technical progress, critical sense of the work performed, confidence in the procedure and reduction of the time needed to perform the anastomosis after 12 weeks practice. In response to the multiplicity of factors that currently influence the cardiovascular surgeon training, there have been combined efforts to reform the practices of surgical medical training.

**CONCLUSION:**

1 - The four models of simulators offer a considerable contribution to the field of cardiovascular surgery, improving the skill and dexterity of the surgeon in training. 2 - Residents have shown interest in training and cooperate in the development of innovative procedures for surgical medical training in the art.

**Table:** 

**Abbreviations, acronyms & symbols**	
BSCVS	= Brazilian Society of Cardiovascular Surgery
CVS	= Cardiovascular surgery
EACTS	= European Association for Cardio-Thoracic Surgery
ICU	= Intensive care unit
OPCABG	= Off-pump coronary artery bypass grafting

## INTRODUCTION

Acquiring competence in cardiac surgery is a complex and multifactorial process, which can take years of experience and training, working hard. The "new paradigm of continuing education in surgery" says, "to provide appropriate educational opportunities and gain knowledge, you must perform the trial skills"^[1]^.

"The surgery simulation plays an increasingly important role in the educational process and serves to bridge the gap in the current training model, which is the operative exposure"^[2]^. It also allows us to assess the cognitive components and expertise of the operator in cardiothoracic surgery. Training in crisis management and staff training have become more present in the care of critically ill patients. The addition of adverse conditions in scenarios enhances the value of the training exercise and provides a method to test responses to emergency situations. The suggested support of interactive and intensive programs will increase the ability of staff to effectively manage the perioperative events of cardiac surgery, simulating specific and global crises in the operating room and intensive care unit (ICU).

As in other surgical specialties, different procedures in cardiac surgery can be divided into sub-areas of expertise. In this sense, different types of simulators may be used, providing the opportunity to develop and evaluate a training program for Resident Medical Doctors.

In particular, how to approach the coronary arteries and the knowledge of anastomoses are fundamental techniques before entering the operating room. Using simulator models, the resident has the opportunity to put into practice their theoretical learning of the literature on coronary surgery requirements.

In response to the myriad of factors that currently influence the training of the cardiovascular surgeon, there have been combined efforts to reform and transform the medical training practices, allowing the resident to repeatedly perform the usual surgical procedures, with the advantage of filling the most important gap in the current training model: Exposure surgery^[3-6]^.

Due to the aforementioned reasons, The European Association for Cardio-Thoracic Surgery (EACTS), concerned with the cardiovascular surgeon training of their institution, focused their efforts on changing the surgical training practices and encouraged the opening of a competition for cardiac surgery trainees to build their own simulator for coronary anastomosis, using everyday materials.

Six simulation prototypes were built by cardiovascular surgery trainees competing for a prize. They were presented in the 25^th^ European Association for Cardio-Thoracic Surgery (EACTS) Annual Meeting on October 1-5, 2011, in Lisbon, Portugal. The overall evaluation of each prototype was performed according to preset development criteria and all simulators provided a considerable contribution to the simulation field in cardiovascular surgery.

The cardiovascular surgery team from Valladolid, Spain, was selected for the "Ethicon Prize" for building a simulator for coronary anastomosis surgery, known as Arroyo simulator^[7]^.

When designing simulation prototypes, trainees have demonstrated their ability to think, which is very important for the development of the future of surgical technique innovation.

In the following EACTS Congress in 2012 and 2013, prototypes for mitral valve repair and aortic surgery were presented, respectively.

### Objective

To assess the technical performance of cardiovascular surgery residents, through systematic training in coronary anastomosis using Arroyo simulator and three different types of UNIFESP simulation models.

To provide adequate educational opportunities to gain the necessary knowledge in the trial of surgical skills.

To maximize the training effect through continuous personalized feedback, provided by the supervising surgeon.

## METHODS

This project was scheduled by the Cardiovascular Surgery Division and developed in the Alpha Center of Health Skills, Undergraduate Rectory, EPM - UNIFESP. The training began in May 2013 and the data collection ended on February 28, 2014.

Ten residents of the Cardiovascular Surgery Division were invited to perform coronary anastomosis on surgical training simulators. Participated in this survey: 1^st^ year residents=3; 4^th^ year=1; 5^th^ year=3; and 6^th^ year=3. This work was also extended to 5^th^ year medical degree students participating in surgical training activities in the Cardiovascular Surgery Division during their 40-weekperiod. Our Division, at that time, did not have 2^nd^ or 3^rd^ year residents.

For teaching purposes, the participants were divided into three groups: Group 1: 1^st^ year residents; Group 2: 4^th^ or 5^th^ year residents; and Group 3: 6^th^ year residents. The 5^th^ year medical degree students participated as observers and initiated practical experience, with only 6 hours of practice, under the supervision of the residents.

The training workload during the first 24 sections (6 months) was 2 hours weekly or 8 hours per month, using only the Arroyo simulator model, followed by 16 sections (2 months) with increased complexity of surgical procedures performing 4 hours weekly or 16 hours per month, using four simulation models.

Supervision of surgical training with simulators was performed by a Cardiac Surgeon in constant observation of activities, changing the systematic training for more complex situations, and trying to assess logical reasoning and residents' performance compared to different situations created at the time.

The Alpha Center for Health Skills - EPM - UNIFESP was used so that the residents' training could take place in a non-sterile simulated operating room environment. The workplace simulating an operating room had all the usual components. The surgery was performed in Arroyo's simulators box type or dummy Unifesp model, which has an opening in the chest cage, with access to mobile cardiopulmonary structures. The training was videotaped, allowing for further analysis of the images in order to assess the applicant's evolution as well as correct technique and systematic surgical procedures performance.

Four simulation models were used to perform the coronary anastomosis technique:

- Arroyo simulator model;
- simulation model with dummy;
- simulation model with dummy and bovine heart;
- simulation model with dummy and pulsatile pig heart.

### I - Arroyo simulator model

**Fig. 1:**
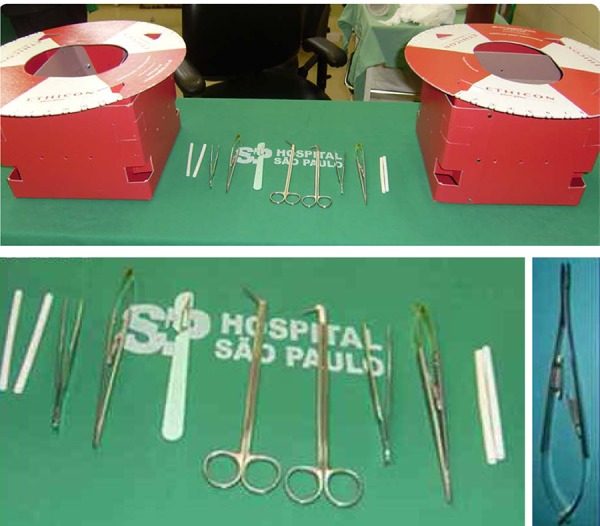
Arroyo box Simulator - Instrumental used for coronary anastomosis training. Featured port: Castroviejo Needle-Holder.

The Arroyo simulator, developed by Heart Surgery trainees from Valladolid, Spain, was featured in the 40^th^ Congress of the Brazilian Society of Cardiovascular Surgery, in Florianópolis in April 2013. Professor Paul Sergeant (Belgium) was responsible for the presentation of the simulator to guest surgeons and the orientation of the coronary anastomosis training. For the simulator training, the Arroyo - Ethicon box, silicone tubing, wire polypropylene (Prolene) 7-0, and Castroviejo needle holder were used (Figure 1).

**Fig. 2:**
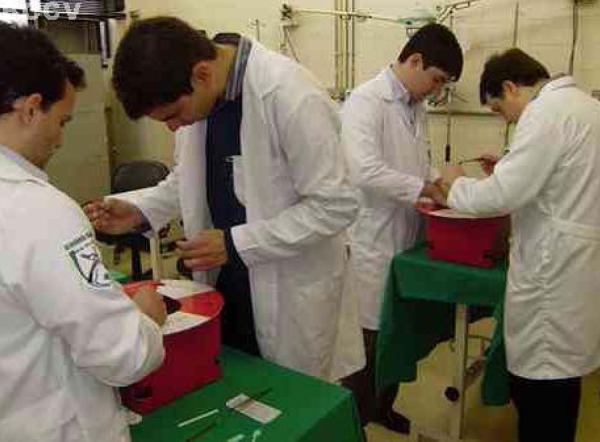
Residents in training using Arroyo simulator.

The training of Residents of the Cardiovascular Surgery Division, EPM - UNIFESP, started in May 2013, remaining active until December 2013. It was composed of 24 training sessions with the Arroyo simulator. Initially, ten residents participated in the activities, grouped in pairs with varying degrees of experience (Figure 2).

**Fig. 3:**
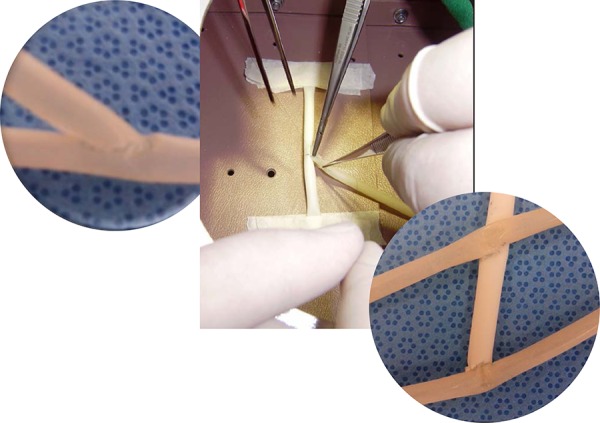
Anastomoses confection with silicone tubes: a- end-toside anastomosis; b- sequential anastomosis.

Working inside the Arroyo simulator box allowed residents to simulate in-depth anastomosis, using silicone tubes of approximately 5mm diameter. Three modalities of anastomoses were performed: end-to-side, end-to-end, and sequential, with polypropylene suture (Prolene) 7-0 (Figure 3).

During the training, changes were introduced in the orientation of the table, using the horizontal, right and left side planes, simulating the approach of coronary arteries in different walls of the heart.

The supervision was performed by the same Cardiac Surgeon, guiding the coronary anastomosis technique. The training time for each working section was 1 hour and 30 minutes to 2 hours.

For the evaluation of residents' performance, we used criteria already standardized and classified in 10 items (Table 1). A score of 1 to 5 points was used to rank each of these items, setting the following correspondence: Poor=1 to 2; Regular=3 to 4; Excellent=5.

**Table 1:** Skill assessment criteria using the Arroyo simulator, including 10 items and using classification score from 1 to 5 points. (Poor=1 to 2; regular=3 to 4; 5=excellent)

Criterion	Poor		Reg		Excel
**Arteriotomy**	1	2	3	4	5
(porcine model: able to identify target, proper use of blade, single groove, centered)					
**Graft Orientation**	1	2	3	4	5
(proper orientation for toe-heel, appropriate start and end-points)					
**Bite appropriate**	1	2	3	4	5
(entry and exit points, number of punctures, even and consistent distance from edge)					
**Spacing appropriate**	1	2	3	4	5
(even spacing, consistent distance from previous bite, too close vs too far)					
**Use of needle holder**	1	2	3	4	5
(finger placement, instrument rotation, facility, needle placement, pronation and supination, proper finger and hand motion, lack of wrist motion)					
**Use of forceps**	1	2	3	4	5
(facility, hand motion, assist needle placement, appropriate traction on tissue)					
**Needle angles**	1	2	3	4	5
(proper angle relative to tissue and needle holder, consider depth of field, anticipating subsequent angles)					
**Needle transfer**	1	2	3	4	5
(needle placement and preparation from stitch to stitch, use of instrument and hand to mount needle)					
**Suture management/tension**	1	2	3	4	5
(too loose vs tight, use of tension to assist exposure, avoid entanglement)					
**Knot tying**	1	2	3	4	5
(adequate tension, facility, finger and hand follow for deep knots)					
Totals					
Grand total					

**Fig. 4:**
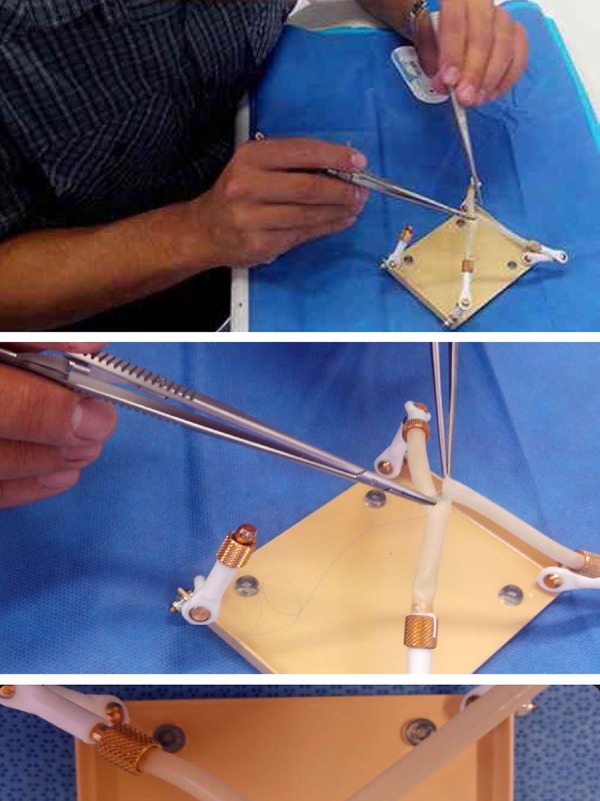
Arroyo simulator for individual coronary anastomosis training. Resident training of coronary anastomosis with individual Arroyo anastomosis simulator.

The best-ranked residents were cast for training with a simulation model, for individual use without assistance, increasing the difficulty of the anastomosis and trying to preserve its technical quality (Figure 4).

**Fig. 5:**
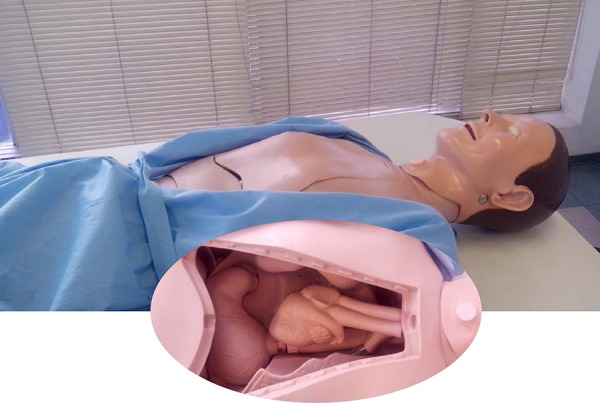
Dummy with removable chest wall. Highlighted, view from inside the chest cavity molds with viscera of the chest and upper abdomen.

In order to create real situations, closer to the surgical environment, a dummy with access to the chest cavity was introduced (Figure 5) and three simulators, UNIFESP's models, were developed, as described below.

### II - Simulation model with dummy and plastic heart

**Fig. 6:**
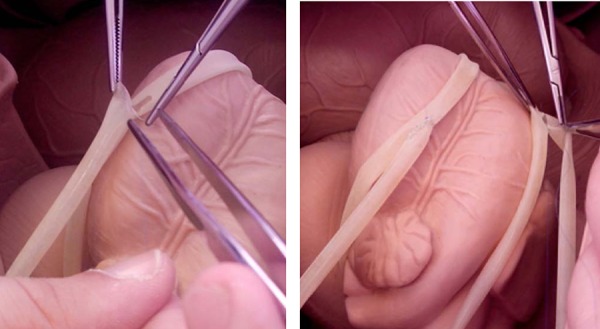
Resident training coronary anastomosis with silicone tubes positioned in the path of the coronary arteries into the chest cavity of the dummy.

Taking advantage of the dummy structure (chest cavity molds with viscera), a plastic heart was used in its anatomical position as a simulator. Silicone tubes were used, positioned and following the anatomical path of the anterior interventricular and right coronary arteries. Using polypropylene suture 7-0, an end-to-side anastomosis was performed (Figure 6).

The patency of the anastomosis test was carried out with saline or dye.

### III - Simulation model with dummy and stationary bovine heart

#### Anatomical piece

The anatomical piece (bovine heart) was obtained by cutting all the vessels, the aorta above the innominate artery, pulmonary artery after the fork, inferior vena cava below the diaphragm, superior vena cava, above the azygous vein and pulmonary veins and as far as possible, the right left atrium.

#### Work inside the dummy

The heart was removed from the freezer 12 hours before the starting time of practice for effective defrosting procedure. Sodium sulfite 2% solution was used as well as coloration to resume the anatomical part.

**Fig. 7:**
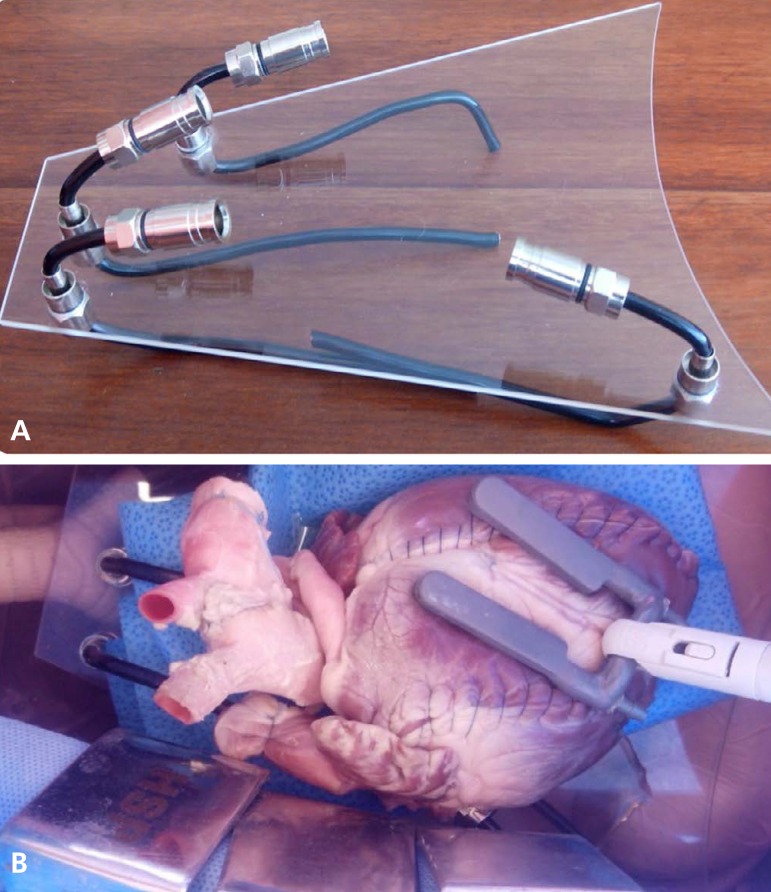
Simulation model with dummy and bovine heart. asupport model with flexible rods; b- bovine heart set on the stand.

To keep the heart in the anatomical position, we have developed an acrylic holder, which fits into the chest cavity of the dummy. It is fixed with four flexible rods and coated copper wires and connected at one end to a cylindrical metal connector. These four rods were fixed onto the vena cava, aorta and pulmonary artery of the heart, to keep it in anatomical position during the surgical training (Figure 7).

The heart set was positioned within the chest cage. The drapes covering the dummy and smaller drapes marked the operating area.

**Fig. 8:**
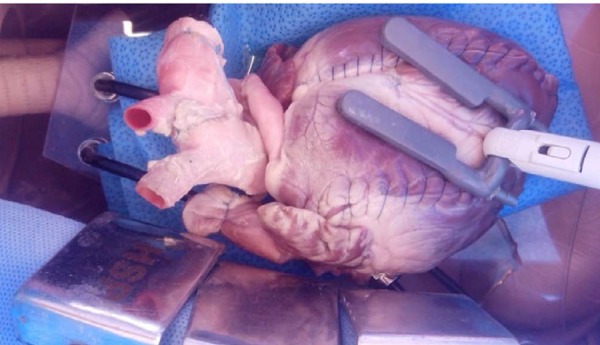
Simulation model with dummy with a bovine heart. Coronary stabilizer and coronary-saphenous vein segment anastomosis performed.

Using coronary stabilizer (Medtronic^®^), the approach to the anterior interventricular coronary artery was performed. An azygous vein segment, obtained from the animal, was used to perform a vena-coronary anastomosis, with a single polypropylene 7-0 suture, and a vena-aortic proximal anastomosis, with a single polypropylene suture 6-0. The coronary artery was 3.0 mm in size, facilitating the procedure. For testing patency of the anastomosis water or dye was used (Figure 8).

### IV - Simulation model with dummy and pulsatile pig heart

In order to arouse interest of the residents and increase the complexity of the training, a simulation model with a dummy and pulsatile pig heart for coronary anastomosis training was designed to simulate a beating heart.

A balloon catheter was introduced by the tip of the left ventricle of the pig heart and externalized by the left atrium. Then, it was connected to the console of an intra-aortic balloon pump (Datascope); and, finally, a softer simulating pulse was connected to monitor.

**Fig. 9:**
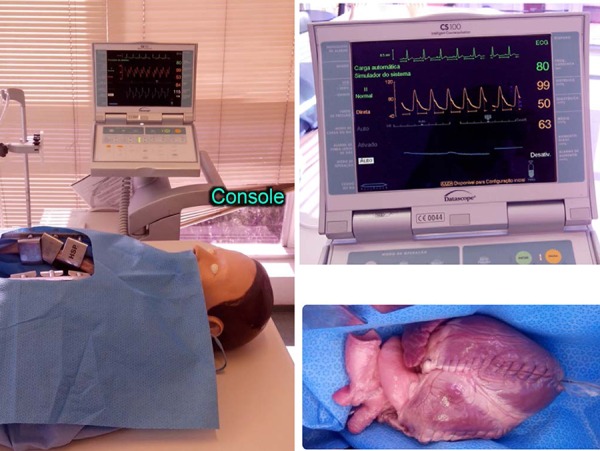
The console of intra-aortic balloon pump (Datascope) with the monitor connected to the simulated pulses and positioned aside the dummy.

The balloon expansion rhythmically moved the atria, ventricles and great vessels (Figure 9).

The pig heart was mounted on its support and it was placed within the chest of the dummy cavity; then, the intra-aortic balloon was initiated, keeping a 70-80 beats per minute pace, simulating the heartbeat and rhythmic movements of the pig heart.

After positioning the coronary stabilizer, the residents proceeded with the approach maneuvers while the heart was still beating. Using azigos vein grafts, the vena-coronary anastomosis was performed with a single 7-0 polypropylene suture. Then, the patency test was conducted using aqueous or dye solution. Finally, the participants performed the vena-aortic proximal anastomosis under tangential clamping of the ascending aorta with a single 6-0 polypropylene suture.

**Fig. 10:**
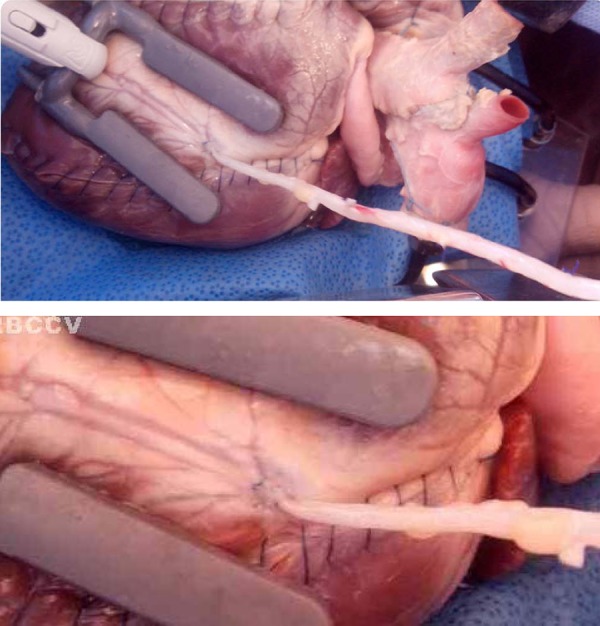
Saphenous vein-coronary artery anastomosis in pig heart using the simulation model with dummy with porcine beating heart.

This simulation model increased the technical difficulties for the resident in training due to the more anatomical features of porcine coronary arteries with a caliber of 1.25 mm in diameter and the presence of pulse induced by the balloon (Figure 10).

## RESULTS

Ten Cardiac Surgery residents from EPM - UNIFESP were invited to attend the training. Due to the varying degrees of experience of the participants, three groups were formed to practice coronary anastomosis. The training was considered completed when the candidate reached 50 points or 90% of the score.

- Ten residents participated in the first four training sessions: Groups 1, 2, and 3.
- Five residents attended the first eight training sessions: Groups 1 and 2.
- Three residents attended 24 training sessions: Group 1.
- Two residents took part in 36 training sessions: Group 1.

The average number of anastomoses performed by residents who participated in 36 sessions was 40 anastomoses each. The classification was based on two parameters: reviewed score in each class and time that it took each group to reach 50 points or 90% of the maximum score.

Out of the ten cardiac surgery residents who were invited to attend the Training Program, only two first year residents completed 36 sessions between May 2013 and March 2014, performing 40 anastomoses each and using the four simulator models, reaching 50 points.

The technical quality of the anastomoses improved with the evolution of the program and through the combination of simulators. There was mastery of technique, skill in handling the surgical instruments and anastomosis surgical performance. Throughout the training, the time taken to perform the anastomosis was shortened by half and the amount of issues in each anastomosis decreased significantly.

The anastomoses were performed with a single 7-0 polypropylene suture and tested with aqueous or red dye solution; the number of additional stitches to contain the leak decreased as experience increased. The types of anastomoses were end-to-side and sequential.

**Fig.11:**
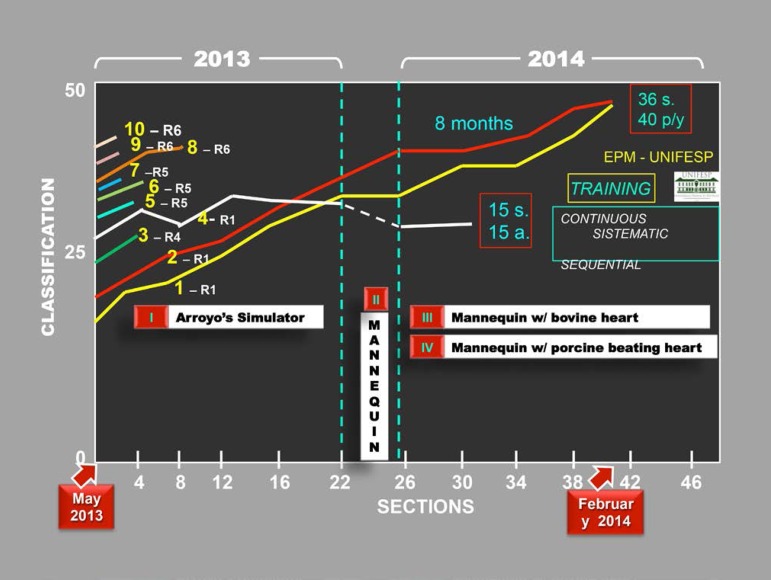
Evolution of First Year Resident Training in Cardiovascular Surgery, with end-to-side coronary anastomosis using the Arroyo simulator.

The two 1^st^ year residents underwent continuous (without interruption), systematic (following the proposed methods) and sequential (attending to a schedule) training, showing a progression in their score, bringing it up to 50 points, set as the maximum score to be reached. The skills and ability in the surgical performance also improved significantly with practice. The learning curve for operating time and the number of events stabilized at about 30 anastomoses. As much as the residents kept training, they had the opportunity to try all simulator models, until they reached the highest score (Figure 11).

The evaluation of performance, skills, dexterity and reasoning ability compared to different difficulties created on purpose showed increasing improvement in the confection of coronary anastomoses.

The use of the Arroyo simulator for 24 sessions facilitated the manipulation of the subsequent three UNIFESP simulation models, increasing participants' expectations for several reasons: 1. It is closer to real-life situation as it uses a porcine beating heart; 2. The coronary arteries had diameters between 1.25 and 1.50 mm, similar to human coronary arteries; and 3. There was the possibility of performing the procedure with the heart beating and testing the use of a coronary stabilizer, simulating myocardial revascularization without cardiopulmonary bypass (off-pump coronary artery bypass grafting - OPCABG).

The use of a pig heart in the simulation of coronary anastomosis surgery allowed for the expansion of this training to other simulation techniques, with an approach to intracardiac structures with or without a heartbeat (Chart 1).

**Chart 1:** Programmed procedures with UNIFESP simulation models.

	1.	Coronary anastomosis
	2.	Tricuspid valve repair
	3.	Tricuspid valve replacement
	4.	Ebstein's anomaly operation
	5.	Right ventricle remodelation surgery
	6.	Blalock-Taussig shunt
	7.	Bidirectional Glenn
	8.	Aortic ring enlargement (Manouguian-Konno)
	9.	Ross operation
	10.	Heart transplant
	11.	Total cavopulmonary anastomosis
	12.	Mitral valve repair
	13.	Mitral valve replacement
	14.	Aortic valve replacement
	15.	ASD Closure
	16.	VSD Closure

ASD=atrial septal defect; VSD=Ventricularseptal defect

## DISCUSSION

Cardiovascular surgery has greater difficulty in adopting new surgical techniques compared to other medical and nonmedical areas. The first generation of cardiac surgeons were brave pioneers, struggling with increasingly complex heart disease and rewarded with the success of their techniques and procedures. Subsequent generations transformed these observations into repeatable processes by establishing routines while enjoying the use of imagination and creativity to develop new techniques or modify already established ones, which contributed to the development of the current cardiovascular surgery, benefiting patients of all ages.

Cardiac surgery has made considerable progress over the last 50 years. However, effective processes that provide active learning and disseminate these advances are missing.

The advances in medical and other instrumental techniques have revolutionized the diagnosis and treatment of heart disease. At the same time, patients have become aware of their own illness, through media and educational methods, and learned how to look after their health.

The competence of the educational system database, now recognized as the most popular, is achieved in the laboratory with the use of simulators. Similar to simulators in the aircraft industry, this concept has become increasingly popular in medical education worldwide.

Excellence Medical Centers have shown significant progress in teaching and learning with simulators, which are increasingly sophisticated. Developing countries such as India enhance training centers with low operating costs, using simple, inexpensive simulators, but with great efficiency.

The technique requires good psychomotor skills development, through regular practice and accompanied by expert suggestions. For each learning situation, different skill components are defined.

The evolution of training methods in surgical technique has caused changes in the traditional curriculum, in which the resident used to learn the surgical technique in the operating room in a stressful atmosphere.

Practical methods to teach more complex surgical techniques were presented to students in the 5^th^ year of Medical School at EPM - UNIFESP, in 1991-1992, facilitating the understanding of surgical correction of heart defects in discussion and awakening the interest of students. This material, prepared in the Operative Technique Course at the Institution, allowed the scientific documentation of techniques such as posterior enlargement of the aortic annulus (Manouguian operation), anterior enlargement of the aortic annulus (Konno operation), remodeling of the right ventricular outflow tract, among others.

The success of this educational model encouraged the professors of our Cardiovascular Surgery Division to increase the demand for virtual medicine model, with simulators, approaching the real-life situations in the operating environment.

On July 23, 1993, this new methodology for teaching and testing surgical techniques in cardiac surgery was presented to the Brazilian Society of Cardiovascular Surgery at the XI Unicor International Symposium, in São Paulo, where six surgical procedures were performed by professors from Unifesp, surgeons and guests, in the following order: Senning operation (Dr. Miguel Maluf), enlargement of the aortic annulus (Dr. Luiz Carlos Bento de Souza), unsupported mitral valve implantation (Dr. Bayard Gontijo), Atrial fibrillation (Dr. Marcelo Jatene), mitral valve repair (Dr. Francisco Gregori), and cardiac transplantation (Dr. João Nelson Rodrigues Branco)^[8]^.

The successful outcome of this Cardiac Surgery Laboratory made it possible to replicate this experience in other National Events, namely: the 3^rd^ Congress of the Society of Cardiovascular Surgery of São Paulo (SCICVESP) - São José do Rio Preto - 1993^[9]^ and the 21^st^ Congress of Cardiac Surgery (BSCVS) - Porto Alegre - 1994^[10]^. The results of this experiment program led to Prof. José Carlos de Andrade's^[11]^ thesis defense. It was also cause for a presentation at an International Congress, The Second World Congress of Pediatric Cardiology and Cardiac Surgery, in Honolulu, Hawaii, 1997^[12]^.

The Brazilian Society of Cardiovascular Surgery (BSCVS) adopted this training model in their last six national congresses, under the name of Hands On, where new specific simulators used for each module as well as ours had been developed by an author for the same purpose as that of our study and used by 408 surgeons^[13]^.

At the time, the scientific documentation through recording tapes allowed the organization of a file of surgical procedures available in our institution, with free access to interested parties to review the technical details of the procedures performed and teach at undergraduate and graduate levels.

After 24 training sessions with the Arroyo simulator, there was considerable progress in manual skills and dexterity of surgical residents. Among the advantages of the simulator, there were ease of transport and the possibility of individual training in any working environment.

The increased complexity of surgical technique training, now with vascular structures of smaller calibers (1.25 to 1.50 mm), and the ability to work in more real-like tissues led us to train with two new simulator models: Bovine and porcine heart.

The adaptation to new models and simulators was facilitated by the continuous, systematic and sequential training conducted with the Arroyo simulator.

Coronary artery bypass surgery without the aid of cardiopulmonary bypass with a beating heart resurfaced with the introduction of minimally invasive techniques and new stabilizers. For this reason, it was important to develop a method for forming surgeons who perform accurate anastomoses, despite the rhythmic motion of the heart, and to develop technical skills in order to get consistent results in this very demanding field of cardiovascular surgery.

Encouraged by the experience of other authors^[14-19]^ with the use of simulators keeping desktop pulsing to mimic the "off pump" surgery, we built a pulsatile simulator at the Alpha Center for Health Skills in order to train coronary anastomosis surgery as well as other intra- and extra-cardiac techniques that compromise the right side of the heart structures while keeping a rhythmic heartbeat.

The training in this type of mobile simulator did not present great difficulties for the 1^st^ year residents in training, who are used to practicing with the Arroyo simulator and two previous UNIFESP models.

The progression of skills in training residents at this time evolved into simulation techniques to correct heart defects on the right side of the heart using the pulsatile model and on the left side of the heart with a stationary heart.

With these simulation models, we can demonstrate the feasibility of setting up a program based on the simulation of complex surgical procedures in a systematic way. The complexity of each level was determined by the objectives of a predefined training checklist and evaluation. In addition, it was developed and validated as specific content of assessment tools for each simulation scenario.

"In the future, the health system will likely follow the example of civil aviation, military and nuclear power plants, making training based on rigorous simulation and evaluation as part of routine education and medical practice"^[20]^.

The Leuven Training Center (Belgium) has effectively employed a multimodal simulation approach, incorporating virtual learning since 2000. This center has trained 950 surgeons and anesthesiologists from 61 countries^[21]^.

Finally, training activities, and specifically the Surgical Division, should be valued and rewarded. Surgeons' training is supposed to encourage academic promotion and leadership positions. Few academic institutions use this criterion, even though their main mission is the education of future professionals.

It is time to invest in our profession, emphasizing the science of learning and establishing the extensive use of safe and effective learning tools in combination with systematic supervision, essential for the future of Cardiovascular Surgery.

## CONCLUSION

The residents' training in Cardiovascular Surgery (CVS) must not be occasional, but a continuous, systematic and sequential process to achieve the desired results.

The four prototype simulators used for CVS Residents' training have been developed at low cost and have increasingly higher fidelity in simulating real-life situations.

Residents in training have shown interest, commitment and engagement in scheduled activities, with increasing improvement in skills, creativity and logical thinking, which are very important for the development of surgical techniques and innovative procedures.

This training was incorporated into the Medical Residency Program of Cardiovascular Surgery at EPM - UNIFESP and may be useful in the preparation of candidates to become Specialists.

We believe that all institutions that have a medical residency program should have mandatory training of their residents, considering the need for an initial investment and low maintenance cost.

**Table:** 

**Authors' roles & responsibilities**			
MAM	Conduct of operations and/or trials; final manuscript approval		
WJG	Analysis and/or data interpretation; final manuscript approval		
AMB	Conduct of operations and/or trials; final manuscript approval		
TCVNA	Conduct of operations and/or trials; final manuscript approval		
ALM	Conduct of operations and/or trials; final manuscript approval		
CCC	Conduct of operations and/or trials; final manuscript approval		
RVSC	Conduct of operations and/or trials; final manuscript approval		
